# Impact of Heart Failure on In-Hospital Outcomes after Surgical Femoral Neck Fracture Treatment

**DOI:** 10.3390/jcm10050969

**Published:** 2021-03-02

**Authors:** Javier Marco-Martínez, José Luis Bernal-Sobrino, Cristina Fernández-Pérez, Francisco Javier Elola-Somoza, Javier Azaña-Gómez, José Luis García-Klepizg, Emmanuel Andrès, Antonio Zapatero-Gaviria, Raquel Barba-Martin, Fernando Marco-Martinez, Jesus Canora-Lebrato, Noel Lorenzo-Villalba, Manuel Méndez-Bailón

**Affiliations:** 1 Internal Medicine Department, Hospital Clínico San Carlos, Universidad Complutense, Instituto de Investigación Sanitaria del Hospital Clínico San Carlos IdISSC, 28040 Madrid, Spain; javiermarco.z@gmail.com (J.M.-M.); javier.azana@gmail.com (J.A.-G.); jgcuenca85@gmail.com (J.L.G.-K.); manuelmenba@hotmail.com (M.M.-B.); 2 Servicio de Control de Gestión, Hospital 12 de Octubre, 28041 Madrid, Spain; jluis.bernal@movistar.es; 3 Servicio de Medicina Preventiva, Hospital Clínico San Carlos, Universidad Complutense, Instituto de Investigación Sanitaria del Hospital Clínico San Carlos IdISSC, 28040 Madrid, Spain; cfernandezp@salud.madrid.org; 4 Fundación Para la Mejora de la Asistencia Sanitaria, Elola Consultores, 28008 Madrid, Spain; javier.elola@imasfundacion.es; 5 Service de Médecine Interne, Diabète et Maladies Métaboliques, Hôpitaux Universitaires de Strasbourg, 67000 Strasbourg, France; emmanuel.andres@chru-strasbourg.fr; 6 Internal Medicine Department, Hospital Universitario de Fuenlabrada, Facultad de Ciencias de la Salud, Universidad Rey Juan Carlos, Alcorcón, 28942 Madrid, Spain; antonio.zapatero@salud.madrid.org (A.Z.-G.); jesuscanoralebrato@gmail.com (J.C.-L.); 7 Internal Medicine Department, Hospital Universitario Rey Juan Carlos, Facultad de Ciencias de la Salud, Alcorcón, Universidad Rey Juan Carlos, 28933 Madrid, Spain; raquel.barba@hospitalreyjuancarlos.es; 8 Traumatology Department, Hospital Clínico San Carlos, Universidad Complutense, Instituto de Investigación Sanitaria del Hospital Clínico San Carlos IdISSC, 28040 Madrid, Spain; fernando.marco@salud.madrid.org

**Keywords:** heart failure, femoral neck fracture, in-hospital outcomes

## Abstract

Background: Femoral neck fracture (FNF) is a common condition with a rising incidence, partly due to aging of the population. It is recommended that FNF should be treated at the earliest opportunity, during daytime hours, including weekends. However, early surgery shortens the available time for preoperative medical examination. Cardiac evaluation is critical for good surgical outcomes as most of these patients are older and frail with other comorbid conditions, such as heart failure. The aim of this study was to determine the impact of heart failure on in-hospital outcomes after surgical femoral neck fracture treatment. Methods: We performed a retrospective study using the Spanish National Hospital Discharge Database, 2007–2015. We included patients older than 64 years treated for reduction and internal fixation of FNF. Demographic characteristics of patients, as well as administrative variables, related to patient’s diseases and procedures performed during the episode were evaluated. Results: A total of 234,159 episodes with FNF reduction and internal fixation were identified from Spanish National Health System hospitals during the study period; 986 (0.42%) episodes were excluded, resulting in a final study population of 233,173 episodes. Mean age was 83.7 (±7) years and 179,949 (77.2%) were women (*p* < 0.001). In the sample, 13,417 (5.8%) episodes had a main or secondary diagnosis of heart failure (HF) (*p* < 0.001). HF patients had a mean age of 86.1 (±6.3) years, significantly older than the rest (*p* < 0.001). All the major complications studied showed a higher incidence in patients with HF (*p* < 0.001). Unadjusted in-hospital mortality was 4.1%, which was significantly higher in patients with HF (18.2%) compared to those without HF (3.3%) (*p* < 0.001). The average length of stay (LOS) was 11.9 (±9.1) and was also significantly higher in the group with HF (16.5 ± 13.1 vs. 11.6 ± 8.7; *p* < 0.001). Conclusions: Patients with HF undergoing FNF surgery have longer length of stay and higher rates of both major complications and mortality than those without HF. Although their average length of stay has decreased in the last few years, their mortality rate has remained unchanged.

## 1. Introduction

Femoral neck fracture (FNF) is a frequent condition with a growing incidence, partly due to aging of the population, which is expected to affect 6.26 million people world-wide by 2050 [1]. Treatment is mainly surgical [2], requires hospitalization, and is associated with significant morbidity and mortality [3].

Early surgery may decrease complications resulting from immobilization, while delays of more than 48 h have been associated with higher 30-day and 1-year mortality [4]. Therefore, it is recommended that FNF should be treated at the earliest stage, during daytime hours, including weekends [5]. However, early surgery shortens the available time for preoperative medical examination (especially cardiac evaluation), which is critical for good surgical outcomes as most of the patients are old, frail, and present other comorbid conditions [6].

In this sense, heart failure is one of the most important comorbid cardiac conditions; there are an estimated 26 million people with heart failure (HF) worldwide [7]. HF is not only the leading non-obstetrical principal diagnosis for hospital admission in Spain but is also a common comorbidity for other causes [8], such as FNF, increasing its morbi-mortality [9]. Risk factors of FNF in Spain have been analyzed [10,11], as well as the costs of its surgical treatment [12] and the effectiveness of the co-management model between orthopedic surgeons and internists [13]. However, as far as we know, there is no data in our field focused on the impact of heart failure on in-hospital outcomes after FNF surgical treatment. The aim of this study was to determine the impact of heart failure on in-hospital outcomes after surgical femoral neck fracture treatment in a large retrospective cohort.

## 2. Methods

### 2.1. Study Design, Data Source, and Patient Population

We performed a retrospective observational study of patients treated for reduction and internal fixation of FNF. Data were obtained from the Minimum Basic Data Set (MBDS), which include information about the demographic characteristics of patients discharged from hospitals in the Spanish National Health System (SNHS), as well as administrative variables related to patient diseases and procedures performed during the episode, coded according to the international classification of diseases, 9th Revision Clinical modification-ICD-9-CM [14].

The study population included patients older than 64 years discharged between 1 January 2007 and 31 December 2015, with FNF as the principal diagnosis (code 820**), who underwent open or closed reduction with internal fixation (79.15 or 79.35, respectively). To improve data quality and consistency, we excluded all episodes lacking records for sex, age, admission date, or principal diagnosis, as well as those with principal or secondary diagnosis of acute myocardial infarction and acute or subacute coronary syndrome.

Selected episodes were classified into two mutually excluding groups, depending whether the patient presented or not a secondary diagnostic of heart failure (HF); codes 402.01, 402.11, 402.91, 404.01, 404.03, 404.11, 404.13, 404.91, 404.93 or 420.** [15]. All the major complications analyzed are detailed in Table 1.

### 2.2. Statistical Analysis

We considered in-hospital mortality and length of stay (LOS), as outcome variables. Considering patient risk of mortality during admission as a combination of individual causes and the quality of the attention given [16], we used a multilevel risk adjustment model, taking into account the patients’ clinical and demographic variables, together with a center-specific effect [17,18,19]. Two models were used; the first one based on the risk mortality level (RML) of the APR-DRG [20]. The second one was based on the methodology developed by the Centers for Medicare and Medicaid Services (CMS) [21]. The first model included age, gender, length of stay, RML, and HF as independent variables. The second model included the variables of the CMS 30-day risk-adjusted mortality for HF, adapting the data model to the MBDS characteristics and grouping secondary diagnostics following the clinical condition categories (CC) proposed by Pope et al. [22], updated yearly by the Agency for Healthcare Research and Quality [23]. To select the variables included in the adjustment models, we used a backward elimination technique. Levels of significance for selecting and eliminating risk factors were *p* < 0.05 and *p* ≥ 0.10, respectively. Model discrimination was assessed by the receiver-operating characteristics (ROC) curve.

The risk-standardized in-hospital mortality rates (RSMRs) were calculated as the ratio of the number of in-hospital deaths predicted on the basis of the hospital’s performance, with its observed case mix at the number of in-hospital deaths expected on the basis of the all-hospitals performance with that hospital’s case mix, multiplied by the all-hospitals unadjusted in-hospital mortality [24]. Accordingly, if the ratio of in-hospital mortality for a specific hospital is higher than the gross mortality rate, then the probability of mortality in that hospital is above the mean rate for the studied hospitals.

To assess the impact of HF on the in-hospital mortality and to control patient selection bias of patients between both groups, we used propensity score matching (PSM) from the risk-adjusted model, according to CMS, with the option of the k-nearest neighbors and a caliper of 0.05 without replacement, obtaining the average effect in the treated (ATT) patients and 95% confidence intervals.

Given the right-skewed nature of the distribution [25], to adjust LOS, we used a Poisson multilevel regression model, considering as risk factors the age and gender of the patient and the APR-DRGs severity of illness and HF as a secondary diagnosis. The expected average LOS was calculated from individual predictions resulting from the adjusted model. The risk-standardized LOS rate (RSLR) was calculated as the ratio between observed and expected average LOS. Temporal trends of RSMR and RSLR during the period of the study was assessed by a Poisson regression model, with the year as the only independent variable. In every model, the incidence rate ratio (IRR) was calculated with 95% confidence intervals. The episodes of the year 2015 were used to analyze the variability of RSMR and RSLR by center characteristics, according to the number of beds [26].

Continuous variables were expressed as a mean (SD), and categorical variables were expressed as numbers and rates. The correlation between continuous variables was analyzed by the Spearman´s Rank Correlation Coefficient (*p*). Student´s *t*-test was used to compare 2 categories and ANOVA, corrected by the Bonferroni test to compare three or more. Categorical variables were compared by the χ^2^ test or Fisher´s exact test. All statistical contrasts were bilateral, and differences were considered significant for *p* < 0.05. Statistical analysis was performed using STATA 13 (StataCorp LLC, College Station, TX, USA) and SPSS 21.0 (IMB, Armonk, NY, USA).

## 3. Results

A total of 234,159 episodes with FNF reduction and internal fixation were obtained from SNHS hospitals during the study period, and 986 (0.42%) were excluded, resulting in a final study population of 233,173 episodes. The clinical and demographic characteristics of the population studied are shown in Table 2. Mean age was 83.7 (±7) years, and 179,949 (77.2%) were women (*p* < 0.001). In the sample, 13,417 (5.8%) had a main or secondary diagnosis of HF (*p* < 0.001). These patients had a mean age of 86.1 (±6.3) years, which was significantly older than the rest (*p* < 0.001). All the major complications studied showed a higher incidence in patients with HF (*p* < 0.001). Unadjusted in-hospital mortality was 4.1%, which was significantly higher in patients with HF (18.2%) compared to those without HF (3.3%) (*p* < 0.001). Average LOS was 11.9 (±9.6), which was also significantly higher in the group with HF (16.5 ± 13.1 vs. 11.6 ± 8.7; *p* < 0.001). There were no statistically significant differences in in-hospital mortality risk in HF patients between open (1.95; CI: 1.8–2.1) and closed reduction with internal fixation (1.97; CI: 1.85–2.11).

**Figure 1:**
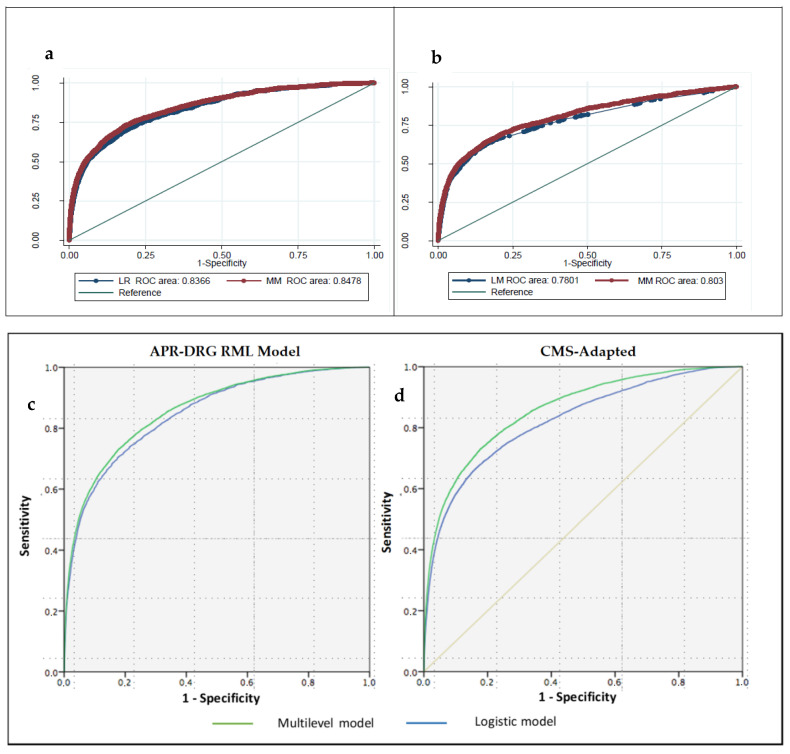
**ROC curves of in-hospital mortality risk adjustment. APR-DRG RML and CMS-adapted models** (**a**) and (**b**) Receiver-operating characteristics (ROC) curves of in-hospital mortality risk adjustment, (**c**) APR-DRG risk mortality level (RML) and (**d**) Centers for Medicare and Medicaid Services (CMS)-adapted models.

Risk-adjusted in-hospital mortality models are displayed in Tables S1 and S2 of the Supplementary Material. Receiver operating characteristics (ROCs) curves are shown in Figure 1. Discrimination was high in both cases, with area under the ROC (AUROC) values = 0.848 (0.837–0.859) in the APR-DRG model and AUROC = 0.803 (0.790–0.816) in the CMS adapted model. The median odds ratio (MOR) was high at1.6 and 1.56, respectively, pointing out a high variability of risk-adjusted outcomes among hospitals.

After including HF as an independent variable in the risk-adjusted models, both showed a significantly high association with in-hospital mortality: OR = 1.97 (1.85–2.11) in the APR-DRG model and OR = 2.97 (2.78–3.17) in the CMS adapted model (*p* < 0.001 in both cases). ATT estimated from PSM further confirmed these results: 0.1822 in episodes presenting HF and 0.116 in those that did not (*p* < 0.05), with a relative risk (RR) of 1.57. The adjusted model for LOS according to APR-DRG severity levels is shown in Table S3 of the Supplementary Material. We found a statistically significant association between HF and LOS (IRR = 1.08; *p* < 0.001), with RSLR of 16.5 days for patients with HF and 11.6 for those without HF.

During the study period, the incidence of FNF reduction and internal fixation increased yearly, from 22,423 to 26,984 episodes without HF and from 1120 to 1885 with HF. No correlation was found between the volume of episodes per center and the RSMR, whether patients had HF or not (*p* > 0.05). In-hospital mortality decreased by 2% every year (IRR = 0.98; *p* < 0.001) in episodes without HF but did not change significantly in the episodes with HF (IRR = 0.996; *p* = 0.688). Average LOS also decreased yearly from 14 ± 10.6 (2007) to 10.4 ± 7.4 days (2015), decreasing from 13.7 ± 10.2 to 10.1 ± 6.9 days in the group without HF and 19 ± 15.6 to 14.9 ± 11.9 days in the HF group. The RSLR did not change during the period (IRR = 0.99), but the HF group showed a statically significant decrease of 3.4% every year (IRR = 0.966) (*p* < 0.001).

Considering only the episodes with HF, in 2015, we only found significant RSMR differences in type 4 hospitals (more than 1000 beds) with an RSMR = 23.3 (±9.5) (*p* < 0.001). In the remaining hospital types, RSMR ranged between 19.0 (±9.6) and 21.1 (±7.8) (*p* > 0.05). RSLR was statically significantly higher in hospitals types 4 (18.8 ± 6.4) and 3 (17.9 ± 6.7) compared to types 2 (15.4 ± 4.4) and 1 (14.3 ± 4.4) (*p* < 0.001).

## 4. Discussion

Patients undergoing FNF reduction and internal fixation with HF were older, predominantly women, developed more severe complications, had a longer stay, and had higher in-hospital mortality rates than patients without HF. During the study period, the annual incidence increased and the average length of stay decreased in both groups. In-hospital mortality rates decreased in patients undergoing FNF reduction and internal fixation without HF, but they did not change significantly in the group with HF; and concerning hospital size, in-hospital mortality rates were higher in hospitals with more than 1000 beds, although there was no correlation between mortality rates and procedure volumes per hospital and we did not find significant differences for the length of stay related with the hospital size.

FNF epidemiology has been widely studied, although population-based research on cardiac complications during surgical hospitalizations for FNF is scarce. In a cohort of 535,745 patients older than 50 years of age, admitted to USA hospitals for FNF between 2012 and 2013, Endo et al. reported a global HF prevalence of 14.9% [27], 7.8% of which were non-surgical cases. The 5.35% HF prevalence in our study may reveal the existence of a remarkable underreporting of secondary diagnoses of HF in the subset of the MBDS that we have analyzed, probably due to poor quality notification of medical comorbidities in surgical discharges. The average age, the percentage of female patients, and the gross mortality rates of our study were similar to those reported by Endo; however, the length of stay was less than half in USA hospitals, which can be explained for notorious differences in clinical management. In both studies, age and HF were strongly associated with in-hospital mortality; in our study, the better discrimination obtained in the risk-adjustment model added a greater reliability to the result.

Several studies on perioperative risk have concluded that HF is an independent predictive factor of perioperative cardiac events [28]. Regarding the surgical treatment of FNF, Cullen et al. [29] assessed the impact of HF in a retrospective observational study with 1116 patients. Their results showed a very high preoperative prevalence of HF (27%), particularly among old patients, with more comorbidities, higher in-hospital mortality, and with an average stay of 10.0 days (higher in HF patients). Sanz-Reig et al. [11] examined a retrospective cohort of 331 patients older than 65 years of age, admitted for FNF between 2011 and 2014 in a Spanish hospital, in which they found an average age of 83 years with 73% of women, a gross mortality rate of 11.4% (higher than that reported in our study), and a prevalence of HF of 19%, also higher than in the general population over 75 years [30]. As in our study, Sanz-Reig et al. identified age and HF as risk factors for in-hospital mortality but did not measure HF impact on other outcomes. Pareja Sierra et al. [31] performed a prospective observational study of 130 patients older than 75 years with osteoporotic FNF, which revealed that suffering from HF was a risk factor for in-hospital mortality and prolonged the length of stay by more than 4 days. Brauer et al. [32] found that the age-adjusted rates of incidence and mortality for FNF in USA decreased from 1985 to 2005, while the comorbidities increased (HF, chronic obstructive pulmonary disease (COPD), and diabetes were the most frequent). Likewise, the trend towards the reduction of the average stay observed in our studies and other studies [33] is parallel to that reported by Nikkel et al. [34], based on patients aged over 50 admitted to hospitals in the State of New York (USA), which went from 12.9 days in 2000 to 5.6 days in 2011.

In our study, LOS average for FNF patients with HF discharged from SNHS hospitals has decreased in recent years, although their mortality risk has not changed significantly. FNF patients with HF have longer hospital stays and higher rates of major complications and mortality than those without HF. Strikingly longer LOS for FNF patients in SNHS hospitals than USA cannot be only related to differences on billing and incentive systems designed for hospitals. Clinical guidelines provide homogeneous and evidence-based approaches for the healthcare management of patients with HF undergoing non-cardiac surgery [35]. HF increases perioperative morbidity and mortality among patients who undergo major non-cardiac surgery [36], and the surgical risk can be minimized through a combination of optimal perioperative medical treatment and the appropriate surveillance and support for the highest risk cases [37]. Our study shows that SNHS hospitals should perform better on FNF patients with HF.

Lizaur-Utrilla et al. evaluated the efficacy of co-management between orthopedic surgeons and internists in the elderly with hip fractures [13]. Their results showed that the patients under this program had similar complication rates, readmissions, and in-hospital mortality to those treated conventionally, but lower mean length of stay. Likewise, Rostagno et al. concluded that the recognition and management of concomitant clinical problems by internists and an integrated perioperative treatment can shorten the length of hospital stays without increasing in-hospital mortality [38]. Given the limited availability of co-management in the NHS, as well as the lack of training and some confusion about the concept of in-hospital consultation [38,39], there seems to still be room for improvement in the care of patients with HF in this context, whose impact should be the subject of further research. In this sense, it is interesting that patients with heart failure are followed by general practitioners since they may have a greater risk of decompensation during the perioperative procedures due to difficulties in managing blood volume when receiving volumetric therapy or red blood cell transfusions, as well as cardiovascular complications (arrhythmias, acute coronary syndrome) in relation to the anesthesia received [37].

Our study has some limitations. The main limitation is due to the low prevalence of HF observed in patients undergoing FNF surgery. This finding can be explained by underreporting of HF in the discharge reports of orthopedic surgery discharges. The inclusion of HF patients in the group without HF partially compensates for the differences found in length of stay, complications, and mortality between both groups, which would go against our hypothesis. Because of this, our outcomes should be interpreted as a lower threshold of the actual effect. The MBDS does not include variables concerning New York Heart Association (NYHA) functional class, natriuretic peptide values, left ventricular ejection fraction, or drugs administered for heart failure during hospitalization. The purpose of our study, in contrast to others [40,41], was not to evaluate the functional situation of the patients but rather the association of the presence of heart failure with the in-hospital mortality and readmission.

The retrospective design and the use of administrative data can be additional limitations. However, the variables obtained from administrative databases have been validated against data from medical records and have been applied to research outcomes on health services [20]. Likewise, although the existence of confounding factors that may not be adequately reflected in the specified models is inherent in the risk adjustment, the time period of this study, the size of the population studied, and the good discrimination of the risk-adjustment models help to overcome this limitation.

## 5. Conclusions

Patients with HF undergoing FNF surgery have longer length of stay and higher rates of both major complications and mortality than those without HF. Although their average length of stay has decreased in the last few years, their mortality rate has remained unchanged. The current low presence of a systematic co-management model in the SNHS implies that there is still room for improvement in the perioperative management of HF patients. Correct reporting of HF in discharge reports of orthopedic surgery discharges is one step towards improving results in this field.

## Figures and Tables

**Table 1:** Major complications studied.

Secondary Diagnosis	CIE-9-MC Codes
Acute Myocardial Infarction	410.0, 410.1
Acute Pulmonary Edema or Shock	427.41, 427.42, 427.5, 518.4, 518.5, 518.51, 518.52, 518.53, 518.81, 518.82, 518.83, 518.84,785.50, 785.51, 798.0, 798.1, 798.2, 798.9, 799.01, 799.02, 998.01
Stroke	433.01; 433.11; 433.21; 433.31; 433.81; 433.91; 434.01; 434.11; 434.91; 436)
Pulmonary Thromboembolism	415.1, 415.11, 415.12, 415.13, 415.19

**Table 2:** Clinical and demographic characteristics according to the presence of heart failure.

	Non-HF Group		HF Group		Total		*p*
	*n*	%	*n*	%	*n*	%	
Mean age (SD)	83.5	±7.1	86.1	±6.3	83.7	±7	<0.001
Women	169,543	77.2	10,406	77.6	179,949	77.2	0.271
History of PTCA	2215	1.0	181	1.3	2396	1.0	<0.001
History of CABG	1111	0.5	81	0.6	1192	0.5	0.221
Stroke	756	0.3	111	0.8	867	0.4	<0.001
Pulmonary thromboembolism	378	0.2	94	0.7	472	0.2	<0.001
Acute pulmonary edema or cardiogenic shock	5164	2.3	2439	18.2	7603	3.3	<0.001
Chronic atherosclerosis	15,912	7.2	1774	13.2	17,686	7.6	<0.001
Cardio-respiratory failure and shock	5625	2.6	2508	18.7	8133	3.5	<0.001
Valvular and rheumatic heart disease	7608	3.5	1742	13.0	9350	4.0	<0.001
Hypertension	118,243	53.8	7788	58.0	126,031	54.1	<0.001
Renal failure	17,509	8.0	3532	26.3	21,041	9.0	<0.001
COPD	14,278	6.5	1767	13.2	16,045	6.9	<0.001
Pneumonia	31,601	14.4	3298	24.6	34,899	15.0	<0.001
Diabetes Mellitus and complications	52,006	23.7	3753	28.0	55,759	23.9	<0.001
Protein_calorie malnutrition	4501	2.0	630	4.7	5131	2.2	<0.001
Dementia and senility	44,402	20.2	2991	22.3	47,393	20.3	<0.001
Hemiplegia, paraplegia, paralysis, functional disability 67–69, 100–102, 177–178	5715	2.6	348	2.6	6063	2.6	0.961
Peripheral vascular disease	125,538	57.1	9169	68.3	134,707	57.8	<0.001
Severe cancers	2619	1.2	192	1.4	2811	1.2	0.014
Trauma CC154–156, 158–161	8143	3.7	603	4.5	8746	3.8	<0.001
Mayor psychiatric disorders	2319	1.1	149	1.1	2468	1.1	0.544
Chronic liver disease	849	0.4	58	0.4	907	0.4	0.406

Number of episodes (N); percutaneous transluminal coronary angioplasty (PCTA); coronary artery bypass grafting (CABG), chronic obstructive pulmonary disease (COPD).

## Supplementary Materials

The following are available online at https://www.mdpi.com/2077-0383/10/5/969/s1, Table S1: Multilevel risk adjustment model of in-hospital mortality according to APR-DRG Risk Mortality Level, Table S2: Multilevel risk adjustment model of in-hospital mortality according to CMS-adapted model, Table S3: Poisson regression model for adjusting the length of stay.
