# Supplementary material for: Impact of Heart Failure on In-Hospital Outcomes after Surgical Femoral Neck Fracture Treatment
Source: J Clin Med. 2021 Mar 2;10(5):969. doi: 10.3390/jcm10050969 (PMC7957564; doi:10.3390/jcm10050969)
Supplement: Supplementary file 1 [file jcm-10-00969-s001.pdf]

Journal of
Clinical Medicine

MDPI

Supplementary Material

Table S1. Multilevel risk adjustment model of in-hospital mortality according to APR-DRG Risk Mortality Level.

	Odds Ratio	*p*	95% Confidence Interval	
Woman	0.66	<0.001	0.62	0.69
Age (1 year)	1.08	<0.001	1.07	1.09
Lenght of stay (1 day)	1.002	0.064	1.000	1.004
Risk mortality level				
0–1	1			
2	2.58	<0.001	2.42	2.75
3	1.48	<0.001	1.38	1.59
4	7.24	<0.001	6.6	7.95

Table S2. Multilevel risk adjustment model of in-hospital mortality according to CMS-adapted model.

	Odds Ratio	*p*	(95% Confidence Interval)	
Women	0.72	<0.001	0.68	0.76
HF	2.97	<0.001	2.78	3.17
History of PTCA	0.74	0.016	0.59	0.95
History of CABG	0.72	0.067	0.51	1.02
Chronic atherosclerosis	1.26	<0.001	1.16	1.37
Cardio-respiratory failure and shock	10.72	<0.001	10.04	11.43
Valvular and rheumatic heart disease	1.26	<0.001	1.15	1.39
Hypertension	0.63	<0.001	0.57	0.70
Stroke	7.71	<0.001	6.32	9.41
Renal failure	3.45	<0.001	3.25	3.66
COPD	1.40	<0.001	1.32	1.49
Pneumonia	0.86	<0.001	0.81	0.91
Diabetes Mellitus and complications	0.81	0.006	0.69	0.94
Protein_calorie malnutrition	1.09	0.003	1.03	1.15
Hemiplegia,paraplegia,paralysis,functional disability 67-69, 100-102,177-178	0.80	0.004	0.69	0.93
Peripheral vascular disease	1.33	<0.001	1.20	1.48
Severe cancers	3.56	<0.001	3.12	4.07
Trauma CC154-156, 158-161	1.44	<0.001	1.29	1.60
Chronic liver disease	3.38	<0.001	2.69	4.25

HF, heart failure; PCTA, percutaneous transluminal coronary angioplasty; CABG, coronary artery bypass grafting; COPD, chronic obstructive pulmonary disease.

Table S3. S3. Poisson regression model for adjusting the length of stay.

	IRR	*p*	(95% Confidence Interval)	
heart failure (HF)	1.08	<0.001	1.06	1.09
Woman	0.99	0.008	0.98	1.00
Age (1 year)	1.08	<0.001	1.07	1.09
Lenght of stay (1 day)	0.999	<0.001	0.999	1.000
Risk mortality level				
0–1	1			
2	1.19	<0.001	1.18	1.19
3	1.66	<0.001	1.64	1.68
4	2.23	<0.001	2.17	2.29
